# Anti-Ebola therapy for patients with Ebola virus disease: a systematic review

**DOI:** 10.1186/s12879-019-3980-9

**Published:** 2019-05-02

**Authors:** James S. Lee, Neill K. J. Adhikari, Henry Y. Kwon, Koren Teo, Reed Siemieniuk, François Lamontagne, Adrienne Chan, Sharmistha Mishra, Srinivas Murthy, Peter Kiiza, Jan Hajek, Elhadj I. Bah, Marie-Claire Lamah, Raymond Kao, Robert A. Fowler

**Affiliations:** 1 Interdepartmental Division of Critical Care Medicine, University of Toronto, Toronto, ON Canada; 2 Department of Critical Care Medicine, Sunnybrook Health Sciences Centre and Interdepartmental Division of Critical Care Medicine and Institute for Health Policy, Management, and Evaluation, University of Toronto, Toronto, ON Canada; 3 Duke University Medical Center, Durham, NC USA; 4 Canadian Forces Health Services Group (CFHS), Toronto, ON Canada; 5 Department of Health Research Methods, Evidence, and Impact, McMaster University, Hamilton, ON Canada; 6 Centre de recherche du CHUS de Sherbrooke and Department of Medicine, Division of Critical Care Medicine, Université de Sherbrooke, Sherbrooke, QC Canada; 7 Division of Infectious Diseases, Sunnybrook Health Sciences Centre and University of Toronto, Toronto, ON Canada; 8 Li Ka Shing Knowledge Institute and Division of Infectious Diseases, St. Michael’s Hospital and University of Toronto, Toronto, ON Canada; 9 Department of Paediatrics, University of British Columbia, Vancouver, BC Canada; 10 Department of Critical Care Medicine, Sunnybrook Health Sciences Centre, University of Toronto, Toronto, ON Canada; 11 Division of Infectious Diseases, University of British Columbia, Vancouver, BC Canada; 12 Ministère de la santé, Conakry, Guinea; 13 Service de la pédiatrie, l’Hôpital Régional de Kindia, Kindia, Guinea; 14 Division of Critical Care Medicine, Western University, London, ON Canada

**Keywords:** Ebola, Drug therapy, Systematic review

## Abstract

**Background:**

Management of Ebola virus disease (EVD) has historically focused on infection prevention, case detection and supportive care. Several specific anti-Ebola therapies have been investigated, including during the 2014–2016 West African outbreak. Our objective was to conduct a systematic review of the effect of anti-Ebola virus therapies on clinical outcomes to guide their potential use and future evaluation.

**Methods:**

We searched PubMed, EMBASE, Global Health, Cochrane Library, African Index Medicus, WHOLIS (inception-9 April 2018), and trial registries for observational studies or clinical trials, in any language, that enrolled patients with confirmed EVD who received therapy targeting Ebola virus and reported on mortality, symptom duration, or adverse effects.

**Results:**

From 11,257 citations and registered trials, we reviewed 55 full-text citations, of which 35 met eligibility criteria (1 randomized clinical trial (RCT), 8 non-randomized comparative studies, 9 case series and 17 case reports) and collectively examined 21 anti-Ebola virus agents. The 31 studies performed during the West African outbreak reported on 4.8% (1377/28616) of all patients with Ebola. The only RCT enrolled 72 patients (0.25% of all patients with Ebola) and compared the monoclonal antibody ZMapp vs. standard care (mortality, 22% vs. 37%; 95% confidence interval for risk difference, − 36 to 7%). Studies of convalescent plasma, interferon-β-1a, favipiravir, brincidofovir, artesunate-amodiaquine and TKM-130803 were associated with at least moderate risk of bias.

**Conclusions:**

Research evaluating anti-Ebola virus agents has reached very few patients with EVD, and inferences are limited by non-randomized study designs. ZMapp has the most promising treatment signal.

**Electronic supplementary material:**

The online version of this article (10.1186/s12879-019-3980-9) contains supplementary material, which is available to authorized users.

## Background

The West African Ebola virus disease (EVD) epidemic of 2014 to 2016 resulted in at least 28,616 cases and at least 11,310 deaths [1]. Case management in prior Ebola virus outbreaks has generally focused on isolating patients with EVD, infection prevention and control procedures, and various degrees of basic supportive care [2]. During the West African outbreak, management of EVD involved progressively more advanced supportive care for patients [3–5]. At the onset of the outbreak, mortality was approximately 74% [6], but eventually fell to 31–37% [7, 8], perhaps due to improved early case finding and supportive care. Specific anti-Ebola virus treatment was only rarely available in West Africa. In contrast, among patients evacuated to European and American hospitals who received intensive care and anti-Ebola virus treatments, mortality was 18.5% [3, 9–13].

In prior outbreaks, there was little support to conduct trials, which meant there was limited evidence to guide clinical care during the recent West Africa outbreak. Several anti-Ebola virus treatments had been offered and administered to patients in West Africa, the United States and Europe. Most of these treatments had only recently been developed, not administered as part of a methodologically rigorous study or trial design, and therefore their net effects are unknown. To assess their potential benefit, we systematically reviewed the literature for all clinical studies that included specific anti-Ebola virus treatments administered to patients with EVD and reported on mortality, symptom duration, or adverse effects.

## Methods

There is no published protocol for this review.

### Literature search

With the assistance of a medical librarian, we searched MEDLINE, EMBASE, Global Health, Cochrane Library, African Index Medicus, and World Health Organization Global Index Medicus (WHOLIS) from inception to 9 April 2018. In MEDLINE and EMBASE, controlled vocabulary terms were combined with keywords for EVD and a broad range of study design terms, including but not limited to a sensitive search filter for randomized clinical trials (RCTs) of therapy [14, 15]. Full details of the searches are available in Additional file 1. We also searched Google, Google Scholar, and trial registries’ websites [16–19]. The reference lists of all relevant retrieved manuscripts were screened and hand-searched, and Ebola clinical care experts were consulted to identify any additional studies.

### Selection criteria

We included studies with at least one patient with confirmed EVD of any age who received a therapy specifically targeting Ebola virus itself or its clinical consequences (including blood component-based strategies) and reporting on at least one outcome of interest (mortality, symptom duration after anti-Ebola treatment initiation, and adverse effects of the treatment). Eligible study designs included RCTs, non-randomized single-arm intervention studies (with or without a control group), prospective and retrospective cohort studies, and case reports and case series, without regard to publication language or date. We excluded studies of supportive care therapies that did not specifically target the Ebola virus (e.g. intravenous fluids, electrolyte and metabolic support, and organ-supportive care such as mechanical ventilation and renal replacement), vaccines for EVD primary prevention or post-exposure prophylaxis, and pre-clinical studies (e.g. involving animals or cell lines or computer models) of anti-Ebola treatments.

Two review authors (JSL and HYK), independently and in duplicate, screened titles and abstracts of retrieved citations and independently assessed full-text manuscripts of citations considered potentially eligible by either reviewer. Disagreements were resolved by consensus through discussion with two additional authors (NKJA and RAF).

### Data extraction and quality appraisal

Two review authors (JSL and HYK) independently and in duplicate extracted data, including patient baseline characteristics (age, sex), study methods (design, eligibility criteria, and for RCTs, method of randomization, allocation concealment and blinding), study interventions and co-interventions, and clinical outcomes of interest (mortality at the latest time point available, adverse effects).

Three review authors (JSL, KT, RAF) assessed the risk of bias in the included studies using the Cochrane Collaboration’s Risk of Bias 2.0 tool for RCTs [20] and the ROBINS-I tool for non-randomized intervention and cohort studies [21]; a fourth author (NKJA) verified selected methodological details of these studies.

### Statistical analysis

Where meta-analyses were not possible, we report the results as stated in the included studies. For each anti-Ebola virus treatment, we calculated the number and proportion of patients who died. If warranted on the basis of a sufficient number of trials (greater than or equal to 3) and sufficient clinical and methodological homogeneity, we planned meta-analyses of studies of the same intervention, using risk ratios and 95% confidence intervals (CI) to summarize dichotomous outcomes and weighted or standardized mean differences for continuous outcomes. We planned to assess between-study statistical heterogeneity using the I^2^ measure [22] and to use inverse-variance weighted random-effects models [23] for all meta-analyses to incorporate both between- and within-study variation. We considered *P* ≤ 0.05 (two sided) as statistically significant.

## Results

### Study selection

**Fig. 1:**
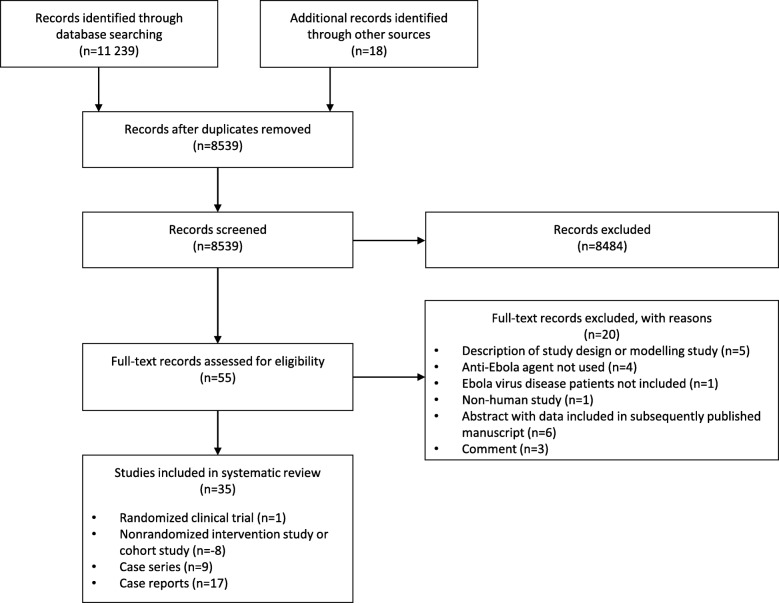
Flow of studies through the systematic review

From 11,257 citations and studies listed in trial registries, 55 studies were selected for full-text review, of which 35 met eligibility criteria (Fig. 1): 1 RCT [24], 8 non-randomized intervention and cohort studies [25–32], 9 case series [11, 33–40], and 17 case reports published in 16 citations [10, 13, 41–54]. These 35 studies collectively examined 21 anti-Ebola agents, including 9 antivirals, 6 blood- or blood component-based therapies, 3 monoclonal antibody treatments, 2 vascular leak syndrome treatments (previously described for other indications [55, 56]), and 1 antimalarial drug, which we included because of the possibility of anti-Ebola virus effects [57]. Thirty-one eligible studies, published in 30 citations, were conducted during the 2014–2016 West African outbreak [10, 11, 13, 24–32, 36–40, 42–54] and reported on 4.8% (1377/28616) of all patients with a diagnosis of EVD.

### RCT and non-randomized studies

Details of the setting, the intervention and control groups from one RCT and 8 non-randomized intervention studies of anti-Ebola therapies are reported in Table 1. The 8 non-randomized intervention studies include 3 single-arm intervention studies with concurrent controls, 3 single-arm intervention studies with historical controls, 1 single-arm uncontrolled intervention study, and 1 retrospective cohort study. The only RCT [24], which examined an anti-Ebola virus monoclonal antibody (ZMapp), enrolled 72 patients (0.25% of all patients with Ebola from the West African outbreak). Although the trial’s risk of bias is low (Table 2), the number of patients enrolled is small and the results are accordingly imprecise (Table 3). Mortality was 22% (8/36) in the ZMapp group and 37% (13/35) in the standard care group. Using Bayesian analysis, the posterior probability that ZMapp was superior to usual care was 91.2%, failing to meet the pre-specified threshold of 97.5%. Frequentist analysis showed a statistically non-significant risk difference of 15% favouring ZMapp (95% confidence interval [CI], − 36% [favouring ZMapp] to 7% [favouring usual care]).

**Table 1:** Randomized trial and non-randomized studies of anti-Ebola therapies

Agent Citation	Setting (n centres)	Intervention group (n included in analysis)	Control group (n included in analysis)
Randomized clinical trial			
ZMapp PREVAIL II Writing group, 2016 [24]	Liberia, Sierra Leone, Guinea, USA (11 centres)	ZMapp 50 mg/kg IV every 3 days (*n* = 36)	Standard care^a^ (*n* = 35)
Non-randomized single-arm intervention study with concurrent controls			
TKM-130803 Dunning et al., 2016 [25]	Port Loko, Sierra Leone (1 centre)	TKM-130803 infusion 0.3 mg/kg IV daily for up to 7 days (*n* = 12)	Standard care^a^ (*n* = 3)
Convalescent whole blood Sahr et al., 2017 [26]	Wilberforce and Hastings, Freetown, Sierra Leone (2 centres)	Convalescent whole blood 450 ml (*n* = 43)	Standard care^a^ (*n* = 25)
Interferon β-1a Konde et al., 2017 [27]^b^	Coyah, Guinea (1 centre for intervention and the majority of control patients)	Interferon-β-1a 30 μg subcutaneously (*n* = 9)	Standard care^a^ (*n* = 38)
Non-randomized single-arm intervention study with historical controls			
Convalescent plasma van Griensven et al., 2016 [28]	Conakry, Guinea (1 centre)	Convalescent plasma 200–250 ml (or 10 ml/kg if < 45 kg); two consecutive transfusions with each unit obtained from a separate convalescent donor (*n* = 84)	Standard care^a^ (*n* = 418)
Favipiravir Sissoko et al., 2016 [29]	Guinea (Conakry, Gueokedou, Macenta, Nzerekore) (4 centres for intervention patients; control patients from Guinea)	Favipiravir (oral) 6000 mg on day 0, then 2400 mg daily on days 1 to 9 (*n* = 111)	Standard care^a^ (*n* = 540) ^c^
Favipiravir Bai et al., 2016 [30]	Jui Town, Sierra Leone (1 centre)	Favipiravir T-705 (oral) 800 mg twice on day 0 and two doses of 600 mg on subsequent days, ranging from 3 to 11 days, until discharge, transfer, or death (*n* = 39)	Standard care (IV fluids limited)^a^ (*n* = 85)
Non-randomized single-arm intervention study without controls			
Brincidofovir Dunning et al., 2016 [31]	Monrovia, Liberia (1 centre)	Brincidofovir (oral) on day 0, 3, 7, 10, and 14 + standard care^a^ (*n* = 4)	None
Retrospective cohort study			
Artesunate-amodiaquine Gignoux et al., 2016 [32]	Foya, Lofa County, Liberia (1 centre)	Artesunate-amodiaquine (*n* = 71)	Artemether-lumefantrine (*n* = 194); no anti-malarial drugs (*n* = 63)

Abbreviations: *USA* United States of America

^a^Standard care and supportive care include any of intravenous fluids, antimicrobial and anti-malarial treatment, electrolyte replacement, medications for symptomatic management, nutritional support, laboratory tests, and hemodynamic monitoring. Medications, laboratory tests, and frequency of hemodynamic monitoring varied among each respective treatment centre

^b^This study uses the term ‘historical controls’, but reports that 21 of the 38 controls were recruited at the same time and in the same centre as patients in the intervention arm [27]. The remaining 17 controls were treated in other centres in Guinea. We therefore classified the study as using concurrent controls

^c^This study presents data on mortality in historical controls in an appendix and used these data to calculate the sample size

**Table 2:** Risk of bias in a randomized trial and non-randomized studies of anti-Ebola therapies

Agent Citation	Bias arising from the randomization process	Bias due to confounding	Bias in selection of participants	Bias in classification of interventions	Bias due to deviations from intended interventions	Bias due to missing outcome data	Bias in outcomes measurement	Bias in selection of results reported	Overall risk of bias
Randomized clinical trial									
ZMapp PREVAIL II Writing Group, 2016 [24]	Low	N/A	N/A	N/A	Some concerns	Low	Low	Low	Low
Non-randomized single-arm intervention study with concurrent controls									
TKM-130803 Dunning et al., 2016 [25]	N/A	Moderate	Moderate	Low	Low	Low	Low	Low	Moderate
Convalescent whole blood Sahr et al., 2017 [26]	N/A	Moderate	Low to moderate	Moderate	Moderate	Low	Low	Low	Moderate
Interferon β-1a Konde et al., 2017 [27]	N/A	Moderate to serious	Low	Moderate to low	Low	Low	Low	Low	Moderate
Non-randomized, single-arm, intervention study with historical control									
Convalescent plasma van Griensven et al., 2016 [28]	N/A	Serious	Moderate	Low	Low	Low	Low	Low	Moderate
Favipiravir Sissoko et al., 2016 [29]	N/A	Serious to critical	Low	Low	Moderate	Low	Low	Low	Moderate
Favipiravir Bai et al., 2016 [30]	N/A	Serious	Moderate	Moderate	Moderate	Moderate	Moderate	Low	Moderate
Non-randomized single-arm intervention study without controls									
Brincidofovir Dunning et al., 2016 [31]	N/A	Critical	Low to moderate	Low	Moderate	Low	Low	Low	Moderate
Retrospective cohort study									
Artesunate-amodiaquine Gignoux et al., 2016 [32]	N/A	Moderate to serious	Moderate	Moderate	Low	Low	Low	Low	Moderate

Abbreviations: *N/A* not applicable

**Table 3:** Design and outcomes in a randomized trial and non-randomized studies of anti-Ebola therapies

Agent Citation	Primary outcome Selected details of design	Dates of study Reason for termination	Mortality	Adverse events
Randomized clinical trial				
ZMapp PREVAIL II Writing group, 2016 [24]	28-day mortality • Adaptive design: plan to update standard-of-care treatment with the investigational drug, if efficacious in interim analysis • All patients recruited in Guinea received favipiravir as standard of care • 6 randomization strata: baseline PCR CT value (≤22 vs. > 22) and location (Liberia/Sierra Leone vs. Guinea vs. USA); in analysis, location strata changed to Liberia/Sierra Leone/USA vs. Guinea • Planned maximum sample size, *n* = 200 (100 per group)	March–November 2015 Trial closed in January 2016 after affected countries declared nearly Ebola free	• 28-day mortality: 22% (8/36), intervention 37% (13/35), control • Bayesian RD − 14% [95% CrI, −34% to 6%] • Bayesian RR 0.62 [95% CrI, 0.29 to 1.24] • Posterior probability that intervention was superior to control, 91.2% (below pre-specified probability threshold of 97.5%)	• Serious adverse events: 31% (11/36), intervention 37% (13/35), control; *p* = 0.62 • One serious adverse event (hypertension) judged to be related to the ZMapp infusion
Non-randomized single-arm intervention study with concurrent controls				
TKM-130803 Dunning et al., 2016 [25]	14-day survival, excluding deaths within 48 h of ETC admission • Concurrent observational cohort for patients who did not meet additional criteria for drug infusion • Plan for randomization of eligible patients to intervention vs. control arm if number of eligible patients exceeded available treatment beds; this scenario did not happen • Planned maximum sample size, *n* = 100	March–June 2015 Study closed after futility boundary reached	• 14-day mortality (intervention): 75% (9/12) 79% (11/14), if the additional 2 patients who died within 48 h are included • Probability of 14-day survival, given 48-h survival, 0.27 [95% CI, 0.06 to 0.58] • 2 of 3 patients in the observational cohort died	One patient had worsening tachypnea within 48 h of the second TKM-130803 infusion; event felt to be compatible with progression of EVD
Convalescent whole blood Sahr et al. 2017 [26]	Not stated; mortality and other outcomes reported • Patients who did not consent to intervention were recruited into control arm • No sample size calculation	December 2014–April 2015 Reason for stopping not stated	• Mortality: 28% (12/43), intervention 44% (11/25), control • One patient who received intervention dropped out and is excluded from the denominator • OR_survival_ with intervention, 2.3 (95% CI, 0.8 to 6.5)	None
Interferon β-1a Konde et al. 2017 [27]	Clearance and/or reduction in viral RNA from day 1 to day 10, as determined by PCR and/or quantitative real time PCR • 21 control patients admitted to the same ETC during the same time period as the treated patients • 17 more control patients selected; they matched treated patients on specified criteria and received care in a Guinean ETC; time period of treatment of these additional controls not stated • ‘sample size of 30–50 chosen to assess feasibility’	March–June 2015 Reason for stopping not stated	• 21-day mortality: 33% (3/9), intervention 84% (32/38), all controls 81% (17/21), controls from same ETC log-rank *p* = 0.026 comparing intervention to 21 controls • Multiple regression models reported OR_mortality_ with intervention, adjusted for CT, 0.13 (*p* = 0.022; CI not reported)	Not reported
Non-randomized, single-arm, intervention study with historical control				
Convalescent plasma van Griensven et al., 2016 [28]	14-day mortality (including deaths from days 3 to 16 after PCR confirmation of EVD) • Control patients treated in the same ETC before the start of the study • Planned sample size, *n* = 260 (130 per group)	February–August 2015 Study closed in July 2015 due to low caseload	• Mortality 3–16 days after diagnosis: 31% (26/84), intervention 38% (158/418), control 34% (30/88), intervention, if 15 patients who also received it are included, of whom 4 died before day 3 • OR_mortality_ with intervention, adjusted for age and CT, 0.88 (95% CI, 0.51 to 1.51)	• No serious adverse events • 8 patients had adverse reactions during or early after the infusion 5 increase in temperature 4 itching or skin rash 1 nausea 2 reactions requiring reduction in infusion rate
Favipiravir Sissoko et al., 2016 [29]	14-day mortality (changed to ‘on-trial mortality’ to include 1 patient who died at day 17) • Control patients (*n* = 540) from database of MSF ETCs in forested Guinea • Initial sample size, *n* = 180 (60 per group defined by age and time of treatment after symptom onset; definition of strata changed to include age and CT)	December 2014–April 2015 Study closed due to low caseload	Mortality: 54% (60/111), intervention 58% (315/540), control 51% (64/126), intervention, if 15 patients who also received it are included, of whom 4 died Adjusted analysis not reported	• Vomiting within 30 min of pill intake occurred in 30 instances (2%) in 21 patients. • No severe adverse events
Favipiravir Bai et al., 2016 [30]	Mortality (time not specified) • Intervention patients treated 1–10 November 2014 • Control patients treated in the same ETC, 10–30 October • Intravenous fluids limited in the study ETC	10 October-10 November 2014 Study closed when research team rotated out of ETC	Mortality: 44% (17/39), intervention 65% (55/85), control [unadjusted *p* = 0.027] Adjusted analysis not reported	Not reported
Non-randomized single-arm intervention study without controls				
Brincidofovir Dunning et al. 2016 [31]	14-day mortality • No control group • Planned maximum sample size, *n* = 140	January 2015 Study closed because manufacturer stopped participation in all studies of brincidofovir for EVD	14-day mortality: 100% (4/4)	• No serious adverse reactions • No serious unexpected serious adverse reactions • Concern that intervention might have contributed to persistent diarrhea in 1 patient
Retrospective cohort study				
Artesunate-amodiaquine Gignoux et al. 2016 [32]	Mortality (time not specified) Cohort study based on natural experiment: 71 patients prescribed artesunate–amodiaquine because of shortage artemether–lumefantrine (given to 194 patients)	June–October 2014 Study closure: not applicable	• Mortality: 50.7% (36/71), artesunate-amodiaquine; 64.4% (125/194), artemether-lumefantrine; 65.1% (41/63), no anti-malaria drugs • Adjusted^a^ RR_mortality_ 0.69 [95% CI 0.54 to 0.89]	Not described

Data are as reported in the primary studies. Abbreviations: *CI* confidence interval, *CrI* credible interval, *CT* cycle time, *ETC* Ebola Treatment Center, *EVD* Ebola virus disease, *IV* intravenous, *MSF* Médecins Sans Frontières, *N/A* Not Applicable, *OR* odds ratio, *PCR* polymerase chain reaction, *RD* risk difference, *RNA* ribonucleic acid, *RR* risk ratio

^a^Adjusted for age, sex, CT value, time from symptom onset to admission, malaria test result, receipt or no receipt of IV fluids, and number of inpatients at the ETC on the day of patient admission

Risk of bias in all non-randomized single-arm intervention studies of convalescent plasma [28], whole blood [26], favipiravir [29], interferon-β-1a [27], artesunate-amodiaquine [32], TKM-130803 [25], and brincidofovir [31] was at least moderate (Table 2). Comparisons of mortality and adverse events between intervention and control arms were limited by non-randomized study designs (Table 3).

In the study of convalescent plasma, the intervention was associated with mortality of 31% (26/84) compared to 38% (158/418) in the historical control group, with an adjusted odds ratio of death of 0.88 (95% CI, 0.51 to 1.51) [28]. Limitations of this study include unknown level of neutralizing antibodies in plasma, small sample size in the intervention group, lack of data on the delivery of co-interventions and supportive care, and inclusion of historical controls. Among patients treated with convalescent whole blood [26], mortality was 28% (12/43) compared to 44% (11/25) in a concurrent standard care group. However, the intervention was not randomly assigned, leading to overall moderate risk of bias from confounding due to baseline and treatment-related differences, in addition to uncertainty in original patient selection criteria.

Favipiravir was investigated in a non-randomized single-arm study (*n* = 126) [29]. Among 99 adult and adolescent patients evaluated, favipiravir was generally well tolerated. However, the lack of a concurrent control group and uncertainty in patient selection criteria leads to moderate risk of bias. In another non-randomized single-arm study, mortality was 44% (17/39) in the favipiravir group and 65% (55/85) in the historical control group; however, the non-random assignment of the intervention, lack of a concurrent control group, and the potential for differential between-group treatments lead again to moderate risk of bias [30].

Interferon β-1a was examined in a non-randomized single-arm study and compared to controls, finding 21-day mortality of 33% (3/9) in the interferon β-1a group and 84% (32/38) in the expanded control group [27]. The non-random assignment of the intervention, lack of a priori sample size calculation, and potential for differential between-group treatments lead to moderate risk of bias.

In a retrospective cohort study of patients with EVD during a period when artemether-lumefantrine was used in an Ebola treatment unit for empiric anti-malarial treatment, mortality was 64% (125/194), compared to 50% (36/71) during a period of drug shortage when artesunate-amodiaquine was used instead [32]. However, there is moderate risk of bias due to the potential for unmeasured residual confounding; in addition, the biological plausibility of artesunate-amodiaquine as an anti-Ebola virus agent is uncertain.

In two non-randomized single-arm studies, TKM-130803 was associated with a 14-day mortality of 75% (9/12) compared 67% (2/3) mortality in the control group [25], and brincidofovir-associated 14-day mortality was 100% (4/4) [31]. The study designs lead to moderate risk of bias.

Given the small number of studies (often only one) of any single intervention and substantial heterogeneity in study design, we did not conduct any meta-analyses.

### Case series and reports

Study details for 26 case series and case reports (reported in 25 publications) are presented in Additional file 1: Table S1, including the number of patients, country of infection, location of clinical care, anti-Ebola virus treatment given, critical care interventions, and clinical outcomes. Duplicate patient descriptions were noted among 13 manuscripts. One case series described patients (some previously reported in other studies) who received care in Europe or the USA [39], and another case series described patients (some previously reported in other studies) who received care in Sierra Leone [40]. Excluding these two cases series, a total of 32 patients receiving anti-Ebola virus therapies were described in case series and case reports, with a mortality of 22% (7/32). Some patients received > 1 anti-Ebola virus agent, including blood-based therapies (*n* = 25), monoclonal antibodies (*n* = 10), antivirals (*n* = 19), and therapies for vascular leak syndrome (*n* = 3).

Among these 26 case series and case reports, 3 case series described 11 patients who received anti-Ebola virus therapies (convalescent whole blood, Virustat [acyclovir], gamma globulin, Marburg convalescent plasma) in Africa prior to the West African EVD outbreak of 2014–2016. An additional report described a patient who received anti-Ebola virus therapies (convalescent plasma, human interferon) in the United Kingdom prior to the West African EVD outbreak. Four reports described 4 patients who received anti-Ebola virus treatment in West Africa (convalescent whole blood, ZMapp, convalescent leukocytes, GS-5743, favipiravir) during the 2014–2016 outbreak. Three case series and 1 case report described 4 patients who initially received anti-Ebola virus therapies in West Africa (convalescent whole blood, ZMapp, TKM-100802) before transfer to the USA. Finally, 3 case series and 1 case report described 5 patients who received anti-Ebola virus therapies solely in the USA, and 8 case reports described 7 patients who received anti-Ebola virus therapies solely in Europe.

Additional file 1: Table S2 presents the number of patients and mortality rates of 18 patients who were treated with an anti-Ebola virus therapy in addition to receiving intensive care. Mortality was 29% (2/7) in patients who received positive pressure ventilation, 25% (6/24) in those who received central venous access or hemodynamic support, 40% (2/5) in those who received renal replacement therapy, 15% (2/13) in those who received non-convalescent blood product transfusions, and 14% (1/7) in those who received parental nutrition. No information about critical care interventions was described in the remaining 14 patients

Additional file 1: Table S3 characterizes the setting, anti-Ebola virus therapy, primary outcome, and status of 9 registered trials of anti-Ebola treatments that have not yet been completed or published as of the search date of April 2018. Crude mortality is described in 9 case series and 17 case reports of anti-Ebola virus treatments (Additional file 1: Table S4)

## Discussion

In this systematic review, we identified 1 RCT, 8 non-randomized single-arm intervention studies and cohort studies, 9 case series and 17 case reports evaluating 21 anti-Ebola virus therapies. The single RCT evaluated the monoclonal antibody ZMapp, had a low risk of bias, and found a statistically non-significant decrease in mortality. All interventions evaluated in non-randomized studies, including convalescent whole blood or plasma, interferon β-1a, favipiravir and the antimalarial artesunate-amodiaquine, were associated with an overall moderate risk of bias, and in some cases serious or critical risk of bias due to confounding, severely limiting inferences regarding treatment effects. The non-randomized evaluations of brincidofovir and TKM-130803 do not provide evidence to support future evaluation.

The strengths of this study include a comprehensive search of published and available non-published clinical literature, triplicate and independent assessment of risk of bias according to the Cochrane framework, and duplicate independent data abstraction. Our review is the first to summarize all such literature in humans with EVD. A previous systematic review included drug screening and pre-clinical studies and fewer clinical studies (*n* = 9) than we included, and focused on identifying existing drugs with potential therapeutic effect [58]. Other reviews provide further information on selected clinical studies of anti-Ebola virus therapies [59, 60], in addition to details of in vitro and animal studies [59].

However, there are many limitations of such a review. We are limited in inferences due to the moderate to serious risk of bias of the majority of studies and the small number of enrolled patients, leading to one RCT and many non-randomized studies all under-powered to detect differences in mortality. The small number of studies (often only one) of any single intervention and important heterogeneity in study design precluded meta-analyses. Several agents with promising pre-clinical findings or case report-based evaluations cannot yet be evaluated for effectiveness based on existing data and study designs. These include the nucleotide analogue prodrug remdesivir (GS-5734), the monoclonal antibody cocktail REGN3470–3471-3479, and the monoclonal antibody MAb114 [61], which in addition to ZMapp and favipiravir, have been evaluated by a World Health Organization-convened independent scientific committee for monitored emergency use of unregistered and investigational interventions, while awaiting additional evidence [62]. Finally, peer review and evaluations of additional therapies may be forthcoming; however, we believe that we are unlikely to have missed any publications of evaluable treatment effects.

Evaluating the effect of treatments on clinical outcomes of patients with EVD is challenging because of its uncommon, periodic, and lethal nature. There have been few pre-existing therapies with strong evidence of potential treatment effect, making prioritization difficult for clinicians, researchers, regulators and funders. Outbreaks have occurred in resource-challenged health systems in West Africa and most recently in DR Congo, Central Africa [63], often in remote areas with delayed recognition of the outbreak’s onset [64, 65]. Small outbreaks may end before any clinical or research response. A historically high mortality rate, including among healthcare workers, leads to diminished clinical capacity for care and research, but also a reluctance to consider using the methodologically strongest RCT design because of the implication that some patients will not receive a potentially beneficial investigational agent. The variable standard of supportive care contributes to a baseline high mortality rate but also makes estimation of treatment effects difficult, possibly leading to selection of patients who are poorly responsive to investigational anti-Ebola virus agents and an inability to compare therapies across studies [6, 66]. Uniform adoption of evidence-based supportive care guidelines [67] in future outbreaks may facilitate the evaluation of anti-Ebola virus therapies.

West African nations most affected in 2014–2016 had not previously experienced an Ebola outbreak, and there was limited pre-existing Ebola-specific clinical and research capacity. With overwhelmed national healthcare systems and a slow international response, there was little opportunity to evolve interventional research programs in parallel with outbreak care. Eventually, early diagnostic and descriptive studies gave rise to an appreciation of the potential impact of supportive and specific EVD therapy [39, 66]. However, among 28,616 infected patients, fewer than 5% had any therapies described or evaluated and only 0.25% participated in a RCT.

This review is a comprehensive summary of data collected on the effects of specific anti-Ebola therapies. Given Ebola’s high mortality rate, sparse treatment options, and high capacity for spread, it is imperative that an adequate research capacity in Ebola outbreak-prone regions be developed and well-supported by the international community. Rigorous prior knowledge synthesis is critical to plan relevant future research. At this stage, the small number of patients exposed to each intervention and design-related limitations preclude strong inferences on clinical effectiveness. However, a better understanding of the paucity of supportive evidence is valuable for various stakeholders. Decision-makers confronted with EVD outbreaks in the future may use these results to prioritize or avoid system-wide delivery of certain experimental interventions. Guideline developers might use the evidence summary to make graded recommendations regarding specific anti-Ebola virus therapies. Lastly, highlighting the insufficiencies of the existing body of evidence could help researchers to design future studies for implementation during an outbreak and to prioritize experimental therapies for future evaluation.

## Conclusions

In this systematic review, we found only one RCT of anti-Ebola virus therapy that was associated with a low risk of bias and a signal of a treatment effect, suggesting that the monoclonal antibody ZMapp should be prioritized for further evaluation in another EVD outbreak. Moderate to serious risk of bias and small sample sizes preclude strong inferences regarding the clinical effects of convalescent whole blood or plasma, favipiravir, interferon β-1a, and the antimalarial artesunate-amodiaquine.

## Additional file

Additional file 1: **Appendix.** Search strategy and supplementary tables. (PDF 575 kb)

## Abbreviations

CI: Confidence Interval
EVD: Ebola virus disease
RCT: Randomized clinical trial
