# Supplementary material for: Anti-Ebola therapy for patients with Ebola virus disease: a systematic review
Source: BMC Infect Dis. 2019 May 2;19:376. doi: 10.1186/s12879-019-3980-9 (PMC6498552; doi:10.1186/s12879-019-3980-9)
Supplement: Supplementary file 1 — Appendix. Search strategy and supplementary tables. (PDF 575 kb) [file 12879_2019_3980_MOESM1_ESM.pdf]

Appendix

Lee et al., *Anti-Ebola therapy for patients with Ebola virus disease: A systematic review*

Literature search strategies

Note that all searches were re-run on 9 April 2018 using the identical terms.

<u>MEDLINE</u>
Database: Ovid MEDLINE(R) In-Process & Other Non-Indexed Citations, Ovid MEDLINE(R) Daily and Ovid MEDLINE(R) <1946 to Present>
Search Strategy:
----
1 hemorrhagic fever, ebola/ or marburg virus disease/
2 (EVD and ebola).mp. [mp=title, abstract, original title, name of substance word, subject heading word, keyword heading word, protocol supplementary concept word, rare disease supplementary concept word, unique identifier]
3 ebola virus disease.mp.
4 Ebola hemorrhagic fever.mp.
5 (ebola adj5 (case* or patient* or treatment* or outcome* or fatality or mortality)).mp.
6 or/1-5
7 exp animals/ not humans.sh.
8 6 not 7
9 exp Animals, Laboratory/
10 exp Animal Experimentation/
11 exp Models, Animal/
12 exp Rodentia/
13 (rat or rats or mouse or mice).ti.
14 or/9-13
15 8 not 14
16 limit 6 to humans
17 15 or 16
18 Epidemiologic Studies/
19 exp Case-Control Studies/
20 exp Cohort Studies/
21 Case control.tw.
22 (cohort adj (study or studies)).tw.
23 Cohort analy$.tw.
24 (Follow up adj (study or studies)).tw.
25 (observational adj (study or studies)).tw.
26 Longitudinal.tw.
27 Retrospective.tw.
28 Cross sectional.tw.
29 Cross-sectional studies/
30 or/18-29
31 17 and 30
32 randomized controlled trial.pt.
33 controlled clinical trial.pt.
34 randomized.ab.
35 placebo.ab.
36 drug therapy.fs.
37 randomly.ab.
38 trial.ab.
39 groups.ab.
40 or/32-39
41 30 or 40

42 17 and 41
43 (dh or dt or pc or rh or rt or su or th).fs.
44 treat*.mp.
45 therap*.mp.
46 intervention*.mp.
47 manag*.mp.
48 (fatality or mortality).mp.
49 or/43-48
50 30 or 40 or 49 (11100006)
51 17 and 50
52 limit 17 to ("therapy (maximizes sensitivity)" or "therapy (maximizes specificity)" or "therapy (best balance of sensitivity and specificity)")
53 51 or 52
54 (Clinical adj (information or characteristics or data or features)).mp.
55 (Fatality rate or Viral load or Virus load or Ct value or ct values or Evd mortality or Case fatality or Fatal outcome or Patients or Presenting or presentation or Survivors or Non-survivors).mp.
56 54 or 55
57 17 and 56
58 53 or 57

<u>EMBASE</u>
Database: Embase <1974 to 2016 July 18>
Search Strategy:
----
1 Ebola hemorrhagic fever/
2 filovirus infection/ or marburg hemorrhagic fever/
3 (EVD and ebola).mp. [mp=title, abstract, heading word, drug trade name, original title, device manufacturer, drug manufacturer, device trade name, keyword]
4 ebola virus disease.mp.
5 hemorrhagic fever, ebola.mp.
6 (ebola adj5 (case* or patient* or treatment* or outcome* or fatality or mortality)).mp.
7 or/1-6
8 exp animals/ or exp invertebrate/ or animal experiment/ or animal model/ or animal tissue/ or animal cell/ or nonhuman/
9 human/ or normal human/ or human cell/
10 8 and 9
11 8 not 9
12 7 not 11
13 animals/ not humans/
14 nonhuman/
15 exp Animal Experiment/
16 exp Experimental Animal/
17 animal model/
18 exp Rodent/
19 (rat or rats or mouse or mice).ti.
20 or/13-19
21 12 not 20
22 limit 21 to human
23 clinical study/
24 case control study/
25 family study/
26 longitudinal study/
27 retrospective study/

28 prospective study/
29 cohort analysis/
30 (Cohort adj (study or studies)).mp.
31 (Case control adj (study or studies)).tw.
32 (follow up adj (study or studies)).tw.
33 (observational adj (study or studies)).tw.
34 (epidemiologic$ adj (study or studies)).tw.
35 (cross sectional adj (study or studies)).tw.
36 or/23-35
37 22 and 36
38 clinical article/
39 exp clinical study/
40 clinical trial/
41 controlled study/
42 randomized controlled trial/
43 major clinical study/
44 double blind procedure/
45 multicenter study/
46 single blind procedure/
47 phase 3 clinical trial/
48 phase 4 clinical trial/
49 crossover procedure/
50 placebo/
51 or/38-50
52 allocat$.mp.
53 assign$.mp.
54 blind$.mp.
55 (clinic$ adj25 (study or trial)).mp.
56 compar$.mp.
57 control$.mp.
58 cross?over.mp.
59 factorial$.mp.
60 follow?up.mp.
61 placebo$.mp.
62 prospectiv$.mp.
63 random$.mp.
64 ((singl$ or doubl$ or trebl$ or tripl$) adj25 (blind$ or mask$)).mp.
65 trial.mp.
66 (versus or vs).mp.
67 or/52-66
68 51 and 67
69 22 and 68
70 37 or 69
71 (dt or fh or th or dm).fs.
72 (treat* or therap* or intervention* or manag* or outcome* or fatality or mortality).mp.
73 71 or 72
74 22 and 73
75 70 or 74
76 limit 22 to ("therapy (maximizes sensitivity)" or "therapy (maximizes specificity)" or "therapy (best balance of sensitivity and specificity)")
77 75 or 76
78 (Clinical adj (information or characteristics or data or features)).mp.

79 (Fatality rate or Viral load or Virus load or Ct value or ct values or Evd mortality or Case fatality or Fatal outcome or Patients or Presenting or presentation or Survivors or Non-survivors).mp.
80 78 or 79
81 22 and 80
82 77 or 81

<u>GLOBAL HEALTH</u>
Database: Global Health <1973 to 2016 Week 27>
Search Strategy:
----
1 ebola.mp.
2 Ebolavirus.od.
3 viral haemorrhagic fevers/
4 or/1-3
5 exp therapy/
6 mortality/ or epidemiology/ or survival/
7 longitudinal studies/ or cohort studies/ or retrospective studies/ or case studies/
8 (case* or patient* or treatment* or outcome* or fatality or mortality or therap*).mp.
9 (Clinical adj (information or characteristics or data or features)).mp.
10 (Fatality rate or Viral load or Virus load or Ct value or ct values or Evd mortality or Case fatality or Fatal outcome or Patients or Presenting or presentation or Survivors or Non-survivors).mp.
11 or/5-10
12 4 and 11

<u>PubMed</u>
Search (((((((case* or patient* or treatment* or outcome* or fatality or mortality or therap*))) OR ((fatality rate OR viral load OR virus load OR ct value OR ct values OR cvd mortality OR case fatality OR fatal outcome OR patients OR presenting OR presentation OR survivors))) OR (((Clinical AND (information or characteristics or data or features)))))) AND ebola) AND ((publisher[sb] OR inprocess[sb] OR pubmednotmedline[sb] OR pubstatusaheadofprint))

<u>Cochrane Library</u>
Date Run: 20/07/16 14:25:20.788
ID Search Hits
#1 ebola:ti,ab,kw (Word variations have been searched)

<u>African Index Medicus</u>

Database : AIM
Search on : ebola
References found : 32

<u>Global Index Medicus</u>
ebola AND (instance:"ghl") AND (db:("WHOLIS" OR "LILACS" OR "WPRIM" OR "IMSEAR" OR "IMEMR" OR "AIM"))

Studies excluded from the systematic review after assessment of full text (n=20)

Description of study design or modelling study (n=5)

1. Edwards T, Semple MG, De Weggheleire A, et al. Design and analysis considerations in the Ebola_Tx trial evaluating convalescent plasma in the treatment of Ebola virus disease in Guinea during the 2014-2015 outbreak. *Clin Trials* 2016; **13**(1): 13-21.
2. van Griensven J, De Weiggheleire A, Delamou A, et al. The Use of Ebola Convalescent Plasma to Treat Ebola Virus Disease in Resource-Constrained Settings: A Perspective From the Field. *Clin Infect Dis* 2016; **62**(1): 69-74.
3. Gutfraind A, Meyers LA. Evaluating large-scale blood transfusion therapy for the current Ebola epidemic in Liberia. *J Infect Dis* 2015; **211**(8): 1262-7.
4. Berry SM, Petzold EA, Dull P, et al. A response adaptive randomization platform trial for efficient evaluation of Ebola virus treatments: A model for pandemic response. *Clin Trials* 2016; **13**(1): 22-30.
5. Bouazza N, Treluyer JM, Foissac F, et al. Favipiravir for children with Ebola. *Lancet* 2015; **385**(9968): 603-4.

Anti-Ebola agent not used (n=4)

1. Bertoli G, Mannazzu M, Madeddu G, et al. Ebola virus disease: Case management in the Institute of Infectious Diseases, University Hospital of Sassari, Sardinia, Italy. *J Infect Dev Ctries* 2016; **10**(5): 537-43.
2. Parra JM, Salmeron OJ, Velasco M. The first case of Ebola virus disease acquired outside Africa. *N Engl J Med* 2014; **371**(25): 2439-40.
3. Kreuels B, Wichmann D, Emmerich P, et al. A case of severe Ebola virus infection complicated by gram-negative septicemia. *N Engl J Med* 2014; **371**(25): 2394-401.
4. Wichmann D, Kreuels B, Schmiedel S, Kluge S. Intensive care treatment of a patient with Ebola virus disease in Germany [German]. *Med Klin Intensivmed Notfmed* 2017; **112**(1): 38-41.

EVD patients not included (n=1)

1. Heald AE, Iversen PL, Saoud JB, et al. Safety and pharmacokinetic profiles of phosphorodiamidate morpholino oligomers with activity against ebola virus and marburg virus: results of two single-ascending-dose studies. *Antimicrob Agents Chemother* 2014; **58**(11): 6639-47.

Non-human study (n=1)

1. Murin CD, Fusco ML, Bornholdt ZA, et al. Structures of protective antibodies reveal sites of vulnerability on Ebola virus. *Proc Natl Acad Sci U S A* 2014; **111**(48): 17182-7.

Abstract with data included in a subsequently published manuscript (n=6)

1. Gignoux E, Azman AS, Ciglenecki I. Artesunate-amodiaquine is associated with reduced Ebola mortality [abstract]. *Trop Med Int Health* 2015; **20**(Suppl 1): 30.
2. Malvy D, JIKI trial Group. Favipiravir in patients with Ebola virus disease [abstract]. *Trop Med Int Health* 2015; **20**(Suppl 1): 45.
3. van Griensven J, on behalf of the Ebola_Tx Consortium. Emergency evaluation of convalescent plasma for Ebola virus disease in Guinea [abstract]. *Trop Med Int Health* 2015; **20**(Suppl 1): 44.
4. Geisen C, Kann G, Strecker T, et al. Manufacture, management and clinical application of pathogen-inactivated Ebola virus convalescent plasma. *Transfus Med Hemother* 2015; **42**(Suppl 1): 28.
5. Sissoko D, Folkesson E, Abdoul M, et al. Favipiravir in Patients with Ebola Virus Disease: Early Results of the JIKI trial in Guinea [abstract]. *Top Antivir Med* 2015; **23**(e-1): 43-4.
6. van Griensven J. Ebola convalescent plasma therapy trials - does it work? [abstract]. *Vox Sang* 2015; **109**(Suppl 1): 69.

Comment (n=3)

1. Kreil TR. Treatment of Ebola virus infection with antibodies from reconvalescent donors. *Emerg Infect Dis* 2015; **21**(3): 521-3.
2. Fedson DS, Jacobson JR, Rordam OM, Opal SM. Treating the Host Response to Ebola Virus Disease with Generic Statins and Angiotensin Receptor Blockers. *MBio* 2015; **6**(3): e00716.

3. Fedson DS, Rordam OM. Treating Ebola patients: a 'bottom up' approach using generic statins and angiotensin receptor blockers. *Int J Infect Dis* 2015; **36**: 80-4.

Supplementary Table 1. Case series and case reports of anti-Ebola therapeutic agents

Author<br>n	Country of infection<br>Location of care	Anti-Ebola agents	Supportive care*	Outcomes	Comments
Case Series					
Dickson et al., 2018 [1]<br><br>n=44	Sierra Leone<br><br>Kerry Town, Sierra Leone	• n=4 convalescent whole blood	• n=38: CVC<br>• n=21, blood products	<u>Mortality</u>: 20/44 (45.5%)<br>• n=3 were evacuated to USA or Europe and survived	This case series includes patients already reported in this table and other patients not individually found in the literature.
Uyeki et al., 2016 [2]<br><br>n=27	Multiple countries<br><br>USA and Europe	• n=23: any investigational therapy (≥1 dose of agents listed)<br>• n=8: ZMapp or ML77<br>• n=6: ZMab<br>• n=5: TKM-Ebola<br>• n=10: favipiravir<br>• n=7:brincidofovir<br>• n=2: FX06<br>• n=10: convalescent plasma<br>• n=1: convalescent whole blood<br>• n=2: amiodarone<br>• n=1: melanocortin	• n=7: MV<br>• n=4: NIV<br>• n=8: vasoactive medications<br>• n=1: transcutaneous pacing<br>• n=5: RRT<br>• n=15: TPN<br>• n=11: PICC<br>• n=18: CVC<br>• n=11: blood products<br>• n=26: IV fluids	<u>28-day mortality</u>: 5/27 (18.5%)<br><br><u>Adverse events:</u><br>• ZMapp or ML77: fever, hypotension, agitation, tachycardia, tachypnea, flushing, palmar pruritus, rash<br>• ZMAb: fever, urticaria, serum sickness<br>• TKM-Ebola: fever, chills, hypotension, SIRS, nausea, lipemia<br>• Favipiravir: nausea, vomiting, elevated AST, neutropenia, QTc prolongation<br>• Brincidofovir: diarrhea, nausea, vomiting, elevated ALT/AST, severe fatigue<br>• Convalescent plasma: TRALI<br>• Amiodarone: bradycardia	This case series includes patients already reported in this table and other patients not individually found in the literature.
Sueblinvong et al., 2015 [3]	Sierra Leone	• TKM-100802 infusion on days 3-8	• CVC<br>• CRRT	Survived and discharged on day 44. Blood Ebola	• TKM-10082 was discontinued because of concern that it was

Author<br>n	Country of infection<br>Location of care	Anti-Ebola agents	Supportive care*	Outcomes	Comments
n=3 (patient 1 described)<br><br>Kraft et al., 2015 [4]<br>n=2 (patient 2 described)†	Transferred to Emory University Hospital, Atlanta, USA on day 4 of illness	• Convalescent plasma on days 8, 9, 11, 12, 14, 15	• MV<br>• Norepinephrine<br>• Hydrocortisone	RNA negative on days 37 and 38.<br><br><u>Adverse event</u>: Transfusion of initial 500 mL of convalescent plasma was associated with worsening dyspnea and increasing oxygen requirements. The patient developed AKI and ARDS, requiring intubation.	contributing to clinical deterioration.<br>• Developed enteropathogenic *Escherichia coli* diarrhea treated with Ceftriaxone.
Sueblinvong et al., 2015 [3]<br><br>n=3 (patient 2 described)	Sierra Leone<br><br>Transferred to University of Nebraska Medical Center, Omaha, USA on day 14	• Sierra Leone: convalescent whole blood (days 9, 13)<br>• Omaha: convalescent plasma; ZMapp given 6 hrs after admission	• MV<br>• CRRT<br>• Multiple vasopressors<br>• Corticosteroids	Died; suspected perforated viscus or other abdominal catastrophe	
Sueblinvong et al., 2015 [3]<br>n=3 (patient 3 described)<br><br>Lidell et al., 2015 [5]<br>n=3 (patient 1 described)‡	Liberia<br><br>Presented to Texas Health Presbyterian Hospital, Dallas, Texas, USA	Brincidofovir on days 11 and 14	• PICC<br>• MV<br>• CRRT<br>• TPN<br>• Norepinephrine<br>• Corticosteroids<br>• non-convalescent frozen plasma	Died on day 15; developed abdominal distention and increased lactate and vasopressor requirements pre mortem.	Initially presented to hospital on day 2 and discharged with azithromycin for sinusitis on day 3. Presented again on day 5 and admitted to ICU on day 6.
Lidell et al., 2015 [5]<br><br>n=3 (patients 2 and 3 described)	USA<br><br>Texas Health Presbyterian Hospital, Dallas, Texas, USA	Patient 2:<br>• Brincidofovir on days 3, 6<br>• Convalescent plasma (2 transfusions)<br>• TKM-Ebola on days 4, 5, 6<br>• ZMapp on day 5	Patient 2:<br>PICC<br><br>Patient 3:<br>No information	Patient 2: survived and discharged on day 15<br><br>Adverse event: ≈6 hrs after TKM-Ebola infusion started, patient developed high fever,	Patients 2 and 3 were nurses in the ICU for patient 1.‡

Author<br>n	Country of infection<br>Location of care	Anti-Ebola agents	Supportive care*	Outcomes	Comments
		Patient 3:<br>• Brincidofovir on day 4<br>• Convalescent plasma (2 transfusions)		rigors, and chills consistent with cytokine release syndrome<br><br>Patient 3: survived and discharged 14 days after fever onset	
Kraft at al., 2015 [4] (patient 1)<br><br>n=2 (patient 1 described)	Liberia<br><br>Transferred to University of Nebraska Medical Center, Omaha, USA on day 8	• TKM-100802 infusion on days 8-14<br>• Convalescent plasma on days 9, 10	• CVC<br>• TPN	Survived and discharged on day 28	
Lyon et al., 2014 [6]<br><br>n=2	Liberia<br><br>Emory University Hospital, Atlanta, USA<br><br>Patient 1 transferred on day 10; Patient 2 transferred on day 14	• Patient 1: convalescent whole blood on day 7; ZMapp on day 9<br><br>• Patient 2: ZMapp on days 10, 13, 16	Patient 1: Whole blood<br><br>Patient 2: CVC, whole blood, platelets	Patient 1: survived and discharged on day 30<br><br>Patient 2: survived and discharged on day 29	Patient 2: malaria positive and prescribed artemether-lumefantrine for 4 days
Mupapa et al., 1999[7] and Guimard et al., 1999 [8]§<br><br>n=8	Democratic Republic of Congo (1995 epidemic in Kikwit)<br><br>Kikwit General Hospital (MSF Belgium), Democratic Republic of Congo	Convalescent whole blood, obtained from 5 survivors of the same outbreak. In 4 blood donors, IgG and IgM Ebola antibodies were detected but Ebola antigen was not detected.	None described	Mortality: 1/8 (12.5%)<br><br>• Patient 1 discharged after 21 days<br>• Patient 2 asymptomatic after 19 days<br>• Patient 3 discharged after 15 days<br>• Patient 4 discharged after 55 days	• During the initial phase of the epidemic, there was little clinical care and the case fatality rate was ≈80%.<br>• By the last phase, HCWs were trained in barrier nursing techniques and there were relatively few patients<br>• These 8 patients were treated during the last phase

Author<br>n	Country of infection<br>Location of care	Anti-Ebola agents	Supportive care*	Outcomes	Comments
				• Patient 5 discharged after 53 days<br>• Patient 6 discharged after 49 days, later developed uveitis<br>• Patient 7 discharged after 49 days	• In 5/6 transfusion recipients, Ebola antigens had disappeared before day 4<br>• In 4/7 recipients, Ebola IgG or IgM antibodies were present before transfusion. After transfusion, IgG and IgM antibodies were detected in 7/8 recipients.<br>• IgM antibodies were never detected in patient 8, who died
Isaacson et al., 1978 [9]<br><br>n=3	Yumbuku Ebola epidemic in northern Zaire in 1976<br><br>Ngaliema Hospital, Kinshasa, Zaire	Patient 2:<br>• Virustat (Acyclovir)<br>• Gamma globulin on days 3, 5<br><br>Patient 3:<br>• Marburg convalescent plasma 2 units (500 ml)	Patient 1: blood transfusion<br><br>Patient 2: entero-vioform for diarrhea; hydrocortisone<br><br>Patient 3: heparin	Patient 1 died on day 8<br><br>Patient 2 died on day 7<br><br>Patient 3 died on day 7	• Ebola was not known at the time of patients 1 and 2<br>• During patient 3's admission, a "Marburg-like" virus was known
Case Reports					
Nicastri et al., 2017 [10] and Chinello et al., 2017 [11]<br><br>n=1	Sierra Leone<br><br>Lazzaro Spallanzani National Institute for Infectious Diseases, Rome, Italy	• Favipiravir 2g load, and 1200 mg twice daily for 10 days<br>• MIL77 50 mg/kg IV (two doses given 3 days apart)	Methylprednisone	Survived and discharged on day 29 of illness	The same patient is reported in Bertoli et. al.,[12] which did not discuss anti-Ebola therapies
Dufour-Gaume et al., 2017 [13]<br><br>n=1	Guinea<br><br>Guinea (French military field hospital)	Favipiravir	Lyophilized plasma transfusions	Survived	
Dörnemann et al., 2017 [14]	Guinea	• ZMapp on days 2, 5, and 8 of life	• Formula milk and routine newborn care	• Survived and discharged on day 33 of life with a weight of	• Mother enrolled in the JIKI trial and self-reported to be 28 weeks of gestational age. She

Author<br>n	Country of infection<br>Location of care	Anti-Ebola agents	Supportive care*	Outcomes	Comments
n=1	Nongo ETC, Conakry, Guinea	• Leukocyte (buffy coat) transfusion from an Ebola survivor (to promote cell-mediated activity) on day 11 of life<br>• GS-5734 on day 19 of life for 12 days	• 5 days of prophylactic ampicillin from birth<br>• Phenobarbitol for clonic muscle activity	3100g. qRT-PCR for Ebola negative in blood, urine, and skin swabs at discharge.<br>• Weekly follow-up to 12 months showed normal neurologic development and growth (15th percentile for age).	delivered a female neonate on day 5 of admission and died.<br>• Neonate (patient) had birth weight of 2800g and estimated to be 35-36 weeks gestational age<br>• CSF was negative for Ebola on day 16
Jacobs et al., 2016 [15]<br><br>n=1	Imported case from Sierra Leone who became symptomatic in UK<br><br>London, UK	Admission 1:<br>• One dose of oral Brincidofovir 200 mg<br>• Two doses of convalescent plasma (300 mL) on consecutive days<br>• ZMAb 50 mg/kg on days 5 and 8 of illness<br><br>Admission 2:<br>• MIL77 on day 5 of<br>• GS-5734 on days 7-21	• CVC<br>• TPN<br>• continuous positive airway pressure<br>• Corticosteroids<br>• Anti-epileptics	Admission 1: survived and discharged from hospital on day 28 with significant fatigue and a hypercoaguable state with thrombocytosis, treated with LMWH and aspirin<br><br>Admission 2: survived and discharged from hospital after 52 days, with residual left leg weakness and left-sided deafness	• The patient was successfully treated for EVD, but relapsed with EVD causing meningoencephalitis<br>• Both CSF and plasma tested positive for Ebola virus RNA 10 months after the initial diagnosis<br>• Infectious virus was recovered from CSF on the day 4, but not on samples from days 16 or 24. No infectious virus was detected in any blood samples.
Palich et al., 2016 [16]<br><br>n=1	Guinea<br><br>Alliance for International Medical Action, N'zérékoré ETC, Guinea	Favipiravir from day 3-17	None described	Survived and discharged on day 22<br><br>Symptom duration after anti-Ebola agent initiation: 19 days	This patient was part of the JIKI trial. The protocol stipulated a 10-day course of favipiravir, but it was decided to continue the drug until the CT corresponded to the limit of virus detection (day 14 of treatment and day 17 of illness).
Petrosillo et al., 2015 [17]	Sierra Leone	• Favipiravir<br>• Convalescent plasma	• MV<br>• CVC	Survived and discharged 39 days after admission	• High Ebola viral load found in bronchial aspirate

Author<br>n	Country of infection<br>Location of care	Anti-Ebola agents	Supportive care*	Outcomes	Comments
and Geisen et al., 2016 [18]¶<br><br>n=1	Transferred to Lazzaro Spallanzani National Institute for Infectious Diseases, Rome, Italy on day 10	• Melanocortin<br>• ZMAb	• TPN<br>• Norepinephrine<br>• Corticosteroids		• Co-infection with *Plasmodium vivax*<br>• Convalescent plasma obtained from an EVD survivor who received care in Germany
Schibler et al., 2015 [19]<br><br>n=1	Sierra Leone<br><br>Kerry Town, Sierra Leone, then transferred to Geneva University Hospital, Geneva, Switzerland on day 5	• ZMAb infusion on days 5, 8<br>• Favipiravir	• CVC<br>• TPN<br>• FFP<br>• Platelets<br>• Heparin	Survived and discharged on day 19	ZMAb differs from ZMapp in replacement of c1H3 with another chimeric monoclonal antibody (c13C6) and a different manufacturing process.
Nicholson-Roberts et al., 2015 [20]<br><br>n=1	n=1<br><br>Sierra Leone<br><br>Kerry Town, Sierra Leone	2 units of convalescent whole blood (500 mL) over 2 days	• CVC<br>• PRBCs<br>• FFP<br>• cryoprecipitate<br>• platelets	Survived and discharged on day 16	
Florescu et al., 2015 [21]<br><br>n=1	Liberia<br><br>Transferred to Nebraska Biocontainment Unit, Omaha, Nebraska, USA on day 6 of illness	• Brincidofovir 200 mg orally on day 6, then 100 mg orally on days 9, 13, 16<br>• 3 units of convalescent plasma on day 8	• CVC<br>• TPN	Survived and discharged home on day 20	
Wolf et al., 2015[22] and Buttner et al., 2014 [23]∏<br><br>n=1	Sierra Leone<br><br>Transferred to University Hospital, Frankfurt, Germany on day 6	• Favipiravir on days 8, 9<br>• FX06 on days 11, 12, 13<br>• Lectin affinity Plasmapharesis on day 13	• NIV then MV<br>• CVC<br>• Arterial line<br>• CRRT then IHD<br>• Norepinephrine	Survived	One of three CRRT waste samples tested positive for Ebola virus RNA (428 copies/mL on day 10; negative on days 18 and 23). Liquid waste was autoclaved prior to disposal.

Author<br>n	Country of infection<br>Location of care	Anti-Ebola agents	Supportive care*	Outcomes	Comments
			• Amiodarone (stopped in Frankfurt)<br>• PRBCs		
Wolf et al., 2015 [22]<br><br>n=1	No information<br><br>Hospital St. Georg, Leipzig, Germany	FX06 on day 11, 12, 13	No information	Died on day 13	This patient is mentioned in the Discussion without full details.
Mora-Rillo et al., 2015 [24]<br><br>n=1	Spain<br><br>La Paz-Carlos III University Hospital, Madrid, Spain	• Convalescent plasma from two donors<br>• Favipiravir	• PICC<br>• TPN<br>• LMWH for deep venous thrombosis<br>• IM triptorelin (gonadotropin-releasing hormone agonist) to stop menstruation	Survived and discharged on day 34. At discharge, the patient had possible sub-acute post-viral thyroiditis.<br><br><u>Adverse event</u>: On day 10, the patient developed ARDS (possibly TRALI), managed without MV.	The same patient is reported in Parra et al.[25] (anti-Ebola agents not discussed in the report)
Connor et al., 2015 [26]†<br><br>n=1	Sierra Leone<br><br>Transferred to Emory University Hospital, Atlanta, USA on day 4	• TKM-100802 infusion on days 3-8<br>• Convalescent plasma on days 8, 9, 11, 12, 14, 15	CRRT, then prolonged intermittent RRT	Survived	• CRRT effluent was negative for Ebola by qRT-PCR on days 1, 3, and 9<br>• Paired blood and urine samples on days 1, 3, and 9 demonstrated medium to low viral load (CT 26-36)
Emond et. al., 1977 [27]<br><br>n=1	England<br><br>England	• Human interferon 3 million units intramuscular every 12 hrs for 14 days<br>• Convalescent plasma	Plasmapheresis for 9 days	Survived and recovered over 10 weeks<br><br>Symptom duration after anti-Ebola agent initiation:<br>14 days	• Ebola acquired after needlestick injury while transferring homogenized liver from a guinea-pig infected with Ebola virus<br>• Convalescent plasma was obtained from survivors in Zaire and Sudan

Unless otherwise noted, ‘day’ refers to day of illness and blood products are non-convalescent.

Abbreviations. AKI: acute kidney injury, ALT: alanine aminotransferase, ARDS: acute respiratory distress syndrome, AST: aspartate aminotransferase CRRT: continuous renal replacement therapy, CSF: cerebral spinal fluid, CT: cycle time, CVC: central venous catheter, ETC: Ebola treatment center, EVD: Ebola virus disease, FFP: fresh frozen plasma, HCW: healthcare worker, IHD: intermittent hemodialysis, IV: intravenous, LMWH: low molecular-weight heparin, MSF: Médecins Sans Frontières, MV: mechanical ventilation, NIV: non-invasive ventilation, PICC: peripherally inserted central catheter, PRBCs: packed red blood cells, (q)RT-PCR: (quantitative) reverse transcriptase polymerase chain reaction, RRT: renal replacement therapy, SIRS: systemic inflammatory response syndrome, TPN: total parental nutrition, TRALI: transfusion-related acute lung injury, USA: United States of America

* Includes critical care interventions and selected co-interventions, such as intravenous fluids, antimicrobial and anti-malarial treatment, electrolyte replacement, medications for symptomatic management, nutritional support, laboratory tests, and hemodynamic monitoring. Medications, type of laboratory tests, and frequency of hemodynamic monitoring varied among treatment centres.
† Patient 1 described by Sueblinvong et al. [3], patient 2 described by Kraft et al. [4], and the patient described by Connor et al. [26] are the same.
‡ Patient 3 described by Sueblinvong et al. [3] and patient 1 described by Lidell et al. [5] are the same.
§ The same subset of 8 patients is described by Guimard et al.[8] and Mupapa et al. [7].
¶ The same patient is described by Petrosillo et al. [17] and Geisen et al. [18].
∏ The same patient is described by Wolf et al. [22] and Buttner et al. [23].

Supplementary Table 2. Mortality of patients who received critical care interventions in case series and case reports*

	Mortality, % (deaths/total)
Positive pressure ventilation	***7 patients***
Mechanical ventilation	40 (2/5)
Non-invasive ventilation	0 (0/1)
Positive airway pressure ventilation	0 (0/1)
Vascular access and hemodynamic support	***24 patients***
Central venous catheter	0 (0/9)
Peripherally Inserted Central Catheter	33 (1/3)
Vasoactive medications	40 (2/5)
Hydrocortisone/Steroids	43 (3/7)
Renal Replacement Therapy	***5 patients***
Continuous Renal Replacement Therapy	50 (2/4)
Intermittent Renal Replacement Therapy	0 (0/1)
Non-convalescent Blood products	***13 patients***
Whole blood	33 (1/3)
Packed red blood cells	0 (0/2)
Fresh Frozen Plasma	33 (1/3)
Lyophilized plasma transfusions	0 (0/1)
Platelets	0 (0/3)
Cryoprecipitate	0 (0/1)
Other	
Total Parental Nutrition	14 (1/7)
Plasmapheresis	0 (0/1)

*Excluding the case series by Dickson et al. [1] and Uyeki et al. [2]

Supplementary Table 3. Registered studies of anti-Ebola agents (as of 9 April 2018)

Study title (identifier)<br>Design; sample size	Setting	Anti-Ebola agent	Primary Outcomes	Recruitment status<br>Date of last update
A Phase I/II Pilot Clinical Trial to Evaluate the Efficacy and Safety of Ebola Virus Disease (EVD) Convalescent Plasma (ECP) for Treatment of EVD (NCT02333578)<br><br>Single arm trial; all patients receive intervention; n=70	ELWA-2 Ebola Treatment Unit, Monrovia, Liberia	Convalescent Plasma	Ebola viral load	Unknown<br><br>January 2015
A Prospective, Open Label Observational, Phase 1 Safety Study of Passive Immune Therapy During Acute Ebola Virus Disease Using Transfusion of INTERCEPT Plasma Prepared From Volunteer Donors Who Have Recovered From Ebola Virus Disease (NCT02295501)<br><br>Single arm trial; all patients receive intervention; n=12	• Emory University, Atlanta, USA<br>• University of Nebraska Medical Center, Omaha, USA	Convalescent Plasma	• Proportion of subjects who survive EVD<br>• Proportion of subjects with adverse events<br>• Proportion of subjects with serious adverse events	Enrolling by invitation<br><br>January 2018
Efficacy of Favipiravir Against Severe Ebola Virus Disease (NCT02662855)<br><br>Unblinded RCT; control group receives supportive care; n=77	Sierra Leone	Favipiravir	Case fatality rate	Completed<br><br>January 2016
PREVAIL IV: Double-Blind, Randomized, Two-Phase, Placebo-Controlled, Phase II Trial of GS-5734 to Assess the Antiviral Activity, Longer-Term Clearance of Ebola Virus, and Safety in Male Ebola Survivors With Evidence of Ebola Virus Persistence in Semen (NCT02818582)<br><br>Placebo-controlled RCT; n=120	Liberia	GS-5734	• To compare the antiviral activity over 28 days following the administration of 5 days of IV GS-5734 versus placebo in male Ebola Virus Disease survivors with evidence of Ebola virus in their semen<br>• To compare the effect of 5 days of IV GS-5734 versus placebo on the detection of Ebola RNA in semen of male survivors of Ebola Virus Disease over 24 weeks	Recruiting<br><br>April 2018
Tolerance and Activity Assessment of High Doses of Favipiravir in Male Survivors With Ebola Virus in the Semen (NCT02739477)	Guinea	Favipiravir	Number of patients undergoing grade 3 or 4 clinical or biological adverse events related to Favipiravir	Recruiting<br><br>May 2016

Study title (identifier)<br>Design; sample size	Setting	Anti-Ebola agent	Primary Outcomes	Recruitment status<br>Date of last update
Single arm trial; n=18				
Clinical Study to Assess Efficacy and Safety of Amiodarone in Treating Patients With Ebola (NCT02307591)<br><br>Unblinded RCT; no patients enrolled	Sierra Leone	Amiodarone	All-cause mortality at 10 days	Study withdrawn<br><br>October 2015
Convalescent plasma for early Ebola virus disease in Sierra Leone: an open-label, non-randomized, controlled clinical trial (ISRCTN13990511; PACTR201602001355272)<br><br>Design unclear; study record describes as RCT and non-randomized; n=130 (intervention) and n=100 (control)	Sierra Leone	Convalescent Plasma	All cause mortality at day 14 post intervention	Completed<br><br>May 2016
Investigation on the efficacy and safety of favipiravir in patients who are infected or strongly suspected of being infected with Ebola virus (UMIN000016101)	Japan	Favipiravir	Patient survival at the end of study or at discontinuation	This study has been completed (last update posted November 2017).
Treatment of Ebola virus disease with TCM: a prospective clinical study (ChiCTR-OON-14005558)<br><br>Non-randomized controlled trial; n=30 (intervention) and n=30 (control)	China-Sierra Leone Friendship Hospital, Sierra Leone	Traditional Chinese Medicine	Mortality	Recruiting<br><br>November 2014

Abbreviations. EVD: Ebola virus disease, TCM: Traditional Chinese Medicine, USA: United States of America

Supplementary Table 4. Mortality of patients who received anti-Ebola therapeutic agents

	Mortality, % (deaths/total) in RCT and non-randomized intervention studies	Mortality, % (deaths/total) in case series and case reports*	Mortality, % (deaths/total) in all studies
Antivirals	**175 patients**	**19 patients**	**194 patients**
TKM-130803	75 (9/12)†		75 (9/12)
TKM-100802		0 (0/2)	0 (0/2)
TKM-Ebola		0 (0/1)	0 (0/1)
Favipiravir	54 (60/111)‡<br>44 (17/39)§	0 (0/7)	49 (77/157)
Brincidofovir	100 (4/4)	20 (1/5)	56 (5/9)
GS-5734		0 (0/2)	0 (0/2)
Interferon β-1a	33 (3/9)		33 (3/9)
Human interferon		0 (0/1)	0 (0/1)
Virustat (Acyclovir)		100 (1/1)	100 (1/1)
Blood-based therapies	**127 patients**	**25 patients**	**152 patients**
Convalescent plasma	31 (26/84)¶	10 (1/10)	29 (27/94)
Convalescent whole blood	28 (12/43)∏	18 (2/11)	26 (14/54)
Convalescent leukocytes (buffy coat)		0 (0/1)	0 (0/1)
Marburg virus convalescent plasma		100 (1/1)	100 (1/1)
Lectin affinity plasmapheresis		0 (0/1)	0 (0/1)
Gamma globulin		100 (1/1)	100 (1/1)
Monoclonal antibody therapy	**36 patients**	**10 patients**	**46 patients**
ZMapp	22 (8/36)	20 (1/5)	22 (9/41)
ZMab		0 (0/3)	0 (0/3)
MIL77		0 (0/2)	0 (0/2)
Vascular leak syndrome therapy		**3 patients**	**3 patients**
FX06		50 (1/2)	50 (1/2)
Melanocortin		0 (0/1)	0 (0/1)
Other	**71 patients**	**0 patients**	**71 patients**
Artesunate-Amodiaquine	51 (36/71)		51 (36/71)

RCT, randomized controlled trial (referring to the study of ZMapp).

* Excluding the case series by Dickson et al. [1] and Uyeki et al. [2].
† An additional 2 patients died within 48 hours and were excluded from the primary analysis [28]. If they are included, the mortality risk is 11/14 (79%).
‡ An additional 15 patients received favipiravir and were excluded from the analysis, of whom 4 died. If these deaths are included, the mortality risk is 64/126 (51%) [29].
§ The data are from reference [30].
¶ An additional 15 patients received convalescent plasma and were excluded from the analysis, of whom 4 died before the 3rd day after diagnosis. If these deaths are included, the mortality risk is 30/88 (34%) [31].
∏ One patient who received convalescent whole blood dropped out of the study and is excluded from the denominator [32].

Note: This table presents unweighted proportions with numerators and denominators summed across studies.

References

1. Dickson SJ, Clay KA, Adam M, Ardley C, Bailey MS, Burns DS, Cox AT, Craig DG, Espina M, Ewington I *et al*: **Enhanced case management can be delivered for patients with EVD in Africa: Experience from a UK military Ebola treatment centre in Sierra Leone**. *J Infect* 2018, **76**(4):383-392.
2. Uyeki TM, Mehta AK, Davey RT, Jr., Liddell AM, Wolf T, Vetter P, Schmiedel S, Grunewald T, Jacobs M, Arribas JR *et al*: **Clinical Management of Ebola Virus Disease in the United States and Europe**. *N Engl J Med* 2016, **374**(7):636-646.
3. Sueblinvong V, Johnson DW, Weinstein GL, Connor MJ, Jr., Crozier I, Liddell AM, Franch HA, Wall BR, Kalil AC, Feldman M *et al*: **Critical Care for Multiple Organ Failure Secondary to Ebola Virus Disease in the United States**. *Crit Care Med* 2015, **43**(10):2066-2075.
4. Kraft CS, Hewlett AL, Koepsell S, Winkler AM, Kratochvil CJ, Larson L, Varkey JB, Mehta AK, Lyon GM, 3rd, Friedman-Moraco RJ *et al*: **The Use of TKM-100802 and Convalescent Plasma in 2 Patients With Ebola Virus Disease in the United States**. *Clin Infect Dis* 2015, **61**(4):496-502.
5. Liddell AM, Davey RT, Jr., Mehta AK, Varkey JB, Kraft CS, Tseggay GK, Badidi O, Faust AC, Brown KV, Suffredini AF *et al*: **Characteristics and Clinical Management of a Cluster of 3 Patients With Ebola Virus Disease, Including the First Domestically Acquired Cases in the United States**. *Ann Intern Med* 2015, **163**(2):81-90.
6. Lyon GM, Mehta AK, Varkey JB, Brantly K, Plyler L, McElroy AK, Kraft CS, Towner JS, Spiropoulou C, Stroher U *et al*: **Clinical care of two patients with Ebola virus disease in the United States**. *N Engl J Med* 2014, **371**(25):2402-2409.
7. Mupapa K, Massamba M, Kibadi K, Kuvula K, Bwaka A, Kipasa M, Colebunders R, Muyembe-Tamfum JJ: **Treatment of Ebola hemorrhagic fever with blood transfusions from convalescent patients. International Scientific and Technical Committee**. *J Infect Dis* 1999, **179 Suppl 1**:S18-23.
8. Guimard Y, Bwaka MA, Colebunders R, Calain P, Massamba M, De Roo A, Mupapa KD, Kibadi K, Kuvula KJ, Ndaberey DE *et al*: **Organization of patient care during the Ebola hemorrhagic fever epidemic in Kikwit, Democratic Republic of the Congo, 1995**. *J Infect Dis* 1999, **179 Suppl 1**:S268-273.
9. Isaacson M, Sureau P, Courteille G, Pattyn SR: **Clinical aspects of Ebola virus disease at the Ngaliema hospital, Kinshasa, Zaire, 1976**. In: *Proceedings of an International Colloquium on Ebola Virus Infection and Other Haemorrhagic Fevers held in Antwerp, Belgium, 6-8 December, 1977.* edn. Edited by Pattyn SR. Amsterdam, The Netherlands: Elsevier / North-Holland Biomedical Press; 1978: 22-26.
10. Nicastri E, Brucato A, Petrosillo N, Biava G, Uyeki TM, Ippolito G, INMI's Ebola Team: **Acute rhabdomyolysis and delayed pericardial effusion in an Italian patient with Ebola virus disease: a case report**. *BMC Infect Dis* 2017, **17**(1):597.
11. Chinello P, Petrosillo N, Pittalis S, Biava G, Ippolito G, Nicastri E, INMI Ebola Team: **QTc interval prolongation during favipiravir therapy in an Ebolavirus-infected patient**. *PLoS Negl Trop Dis* 2017, **11**(12):e0006034.
12. Bertoli G, Mannazzu M, Madeddu G, Are R, Muredda A, Babudieri S, Calia G, Lovigu C, Maida I, Contini L *et al*: **Ebola virus disease: Case management in the Institute of Infectious Diseases, University Hospital of Sassari, Sardinia, Italy**. *J Infect Dev Ctries* 2016, **10**(5):537-543.
13. Dufour-Gaume F, Delaune D, Martinaud C, Sailliol A: **Early and repeated use of plasma for the management of Ebola patients: Reflection around a case**. *Transfus Clin Biol* 2017, **24**(1):5-8.
14. Dornemann J, Burzio C, Ronsse A, Sprecher A, De Clerck H, Van Herp M, Kolie MC, Yosifiva V, Caluwaerts S, McElroy AK *et al*: **First Newborn Baby to Receive Experimental Therapies Survives Ebola Virus Disease**. *J Infect Dis* 2017, **215**(2):171-174.
15. Jacobs M, Rodger A, Bell DJ, Bhagani S, Cropley I, Filipe A, Gifford RJ, Hopkins S, Hughes J, Jabeen F *et al*: **Late Ebola virus relapse causing meningoencephalitis: a case report**. *Lancet* 2016, **388**(10043):498-503.
16. Palich R, Gala JL, Petitjean F, Shepherd S, Peyrouset O, Abdoul BM, Kinda M, Danel C, Augier A, Anglaret X *et al*: **A 6-Year-Old Child with Severe Ebola Virus Disease: Laboratory-Guided Clinical Care in an Ebola Treatment Center in Guinea**. *PLoS Negl Trop Dis* 2016, **10**(3):e0004393.
17. Petrosillo N, Nicastri E, Lanini S, Capobianchi MR, Di Caro A, Antonini M, Puro V, Lauria FN, Shindo N, Magrini N *et al*: **Ebola virus disease complicated with viral interstitial pneumonia: a case report**. *BMC Infect Dis* 2015, **15**:432.

18. Geisen C, Kann G, Strecker T, Wolf T, Schuttfort G, van Kraaij M, MacLennan S, Rummler S, Weinigel C, Eickmann M *et al*: **Pathogen-reduced Ebola virus convalescent plasma: first steps towards standardization of manufacturing and quality control including assessment of Ebola-specific neutralizing antibodies**. *Vox Sang* 2016, **110**(4):329-335.
19. Schibler M, Vetter P, Cherpillod P, Petty TJ, Cordey S, Vieille G, Yerly S, Siegrist CA, Samii K, Dayer JA *et al*: **Clinical features and viral kinetics in a rapidly cured patient with Ebola virus disease: a case report**. *Lancet Infect Dis* 2015, **15**(9):1034-1040.
20. Nicholson-Roberts T, Fletcher T, Rees P, Dickson S, Hinsley D, Bailey M, Lamb L, Ardley C: **Ebola virus disease managed with blood product replacement and point of care tests in Sierra Leone**. *QJM* 2015, **108**(7):571-572.
21. Florescu DF, Kalil AC, Hewlett AL, Schuh AJ, Stroher U, Uyeki TM, Smith PW: **Administration of Brincidofovir and Convalescent Plasma in a Patient With Ebola Virus Disease**. *Clin Infect Dis* 2015, **61**(6):969-973.
22. Wolf T, Kann G, Becker S, Stephan C, Brodt HR, de Leuw P, Grunewald T, Vogl T, Kempf VA, Keppler OT *et al*: **Severe Ebola virus disease with vascular leakage and multiorgan failure: treatment of a patient in intensive care**. *Lancet* 2015, **385**(9976):1428-1435.
23. Buttner S, Koch B, Dolnik O, Eickmann M, Freiwald T, Rudolf S, Engel J, Becker S, Ronco C, Geiger H: **Extracorporeal virus elimination for the treatment of severe Ebola virus disease--first experience with lectin affinity plasmapheresis**. *Blood Purif* 2014, **38**(3-4):286-291.
24. Mora-Rillo M, Arsuaga M, Ramirez-Olivencia G, de la Calle F, Borobia AM, Sanchez-Seco P, Lago M, Figueira JC, Fernandez-Puntero B, Viejo A *et al*: **Acute respiratory distress syndrome after convalescent plasma use: treatment of a patient with Ebola virus disease contracted in Madrid, Spain**. *Lancet Respir Med* 2015, **3**(7):554-562.
25. Parra JM, Salmeron OJ, Velasco M: **The first case of Ebola virus disease acquired outside Africa**. *N Engl J Med* 2014, **371**(25):2439-2440.
26. Connor MJ, Jr., Kraft C, Mehta AK, Varkey JB, Lyon GM, Crozier I, Stroher U, Ribner BS, Franch HA: **Successful delivery of RRT in Ebola virus disease**. *J Am Soc Nephrol* 2015, **26**(1):31-37.
27. Emond RT, Evans B, Bowen ET, Lloyd G: **A case of Ebola virus infection**. *Br Med J* 1977, **2**(6086):541-544.
28. Dunning J, Sahr F, Rojek A, Gannon F, Carson G, Idriss B, Massaquoi T, Gandi R, Joseph S, Osman HK *et al*: **Experimental Treatment of Ebola Virus Disease with TKM-130803: A Single-Arm Phase 2 Clinical Trial**. *PLoS Med* 2016, **13**(4):e1001997.
29. Sissoko D, Laouenan C, Folkesson E, M'Lebing AB, Beavogui AH, Baize S, Camara AM, Maes P, Shepherd S, Danel C *et al*: **Experimental Treatment with Favipiravir for Ebola Virus Disease (the JIKI Trial): A Historically Controlled, Single-Arm Proof-of-Concept Trial in Guinea**. *PLoS Med* 2016, **13**(3):e1001967.
30. Bai CQ, Mu JS, Kargbo D, Song YB, Niu WK, Nie WM, Kanu A, Liu WW, Wang YP, Dafae F *et al*: **Clinical and Virological Characteristics of Ebola Virus Disease Patients Treated With Favipiravir (T-705)-Sierra Leone, 2014**. *Clin Infect Dis* 2016, **63**(10):1288-1294.
31. van Griensven J, Edwards T, de Lamballerie X, Semple MG, Gallian P, Baize S, Horby PW, Raoul H, Magassouba N, Antierens A *et al*: **Evaluation of Convalescent Plasma for Ebola Virus Disease in Guinea**. *N Engl J Med* 2016, **374**(1):33-42.
32. Sahr F, Ansumana R, Massaquoi TA, Idriss BR, Sesay FR, Lamin JM, Baker S, Nicol S, Conton B, Johnson W *et al*: **Evaluation of convalescent whole blood for treating Ebola Virus Disease in Freetown, Sierra Leone**. *J Infect* 2017, **74**(3):302-309.
